# Cohort profile: Examining Neighbourhood Activities in Built Living Environments in London: the ENABLE London—Olympic Park cohort

**DOI:** 10.1136/bmjopen-2016-012643

**Published:** 2016-10-25

**Authors:** Bina Ram, Claire M Nightingale, Mohammed T Hudda, Venediktos V Kapetanakis, Anne Ellaway, Ashley R Cooper, Angie Page, Daniel Lewis, Steven Cummins, Billie Giles-Corti, Peter H Whincup, Derek G Cook, Alicja R Rudnicka, Christopher G Owen

**Affiliations:** 1 Population Health Research Institute, St George's, University of London, Cranmer Terrace, London, UK; 2 MRC/CSO Social and Public Health Sciences Unit, University of Glasgow, Glasgow, UK; 3 Centre for Exercise, Nutrition and Health Sciences, University of Bristol, Bristol, UK; 4 Bristol Biomedical Research Unit in Nutrition, Diet and Lifestyle, National Institute for Health Research, Bristol, UK; 5 London School of Hygiene and Tropical Medicine, London, UK; 6 McCaughey VicHealth Community Wellbeing Unit, NHMRC Centre for Research Excellence in Healthy Liveable Communities, School of Population and Global Health, University of Melbourne, Melbourne, Victoria, Australia

**Keywords:** EPIDEMIOLOGY, PUBLIC HEALTH, SOCIAL MEDICINE

## Abstract

**Purpose:**

The Examining Neighbourhood Activities in Built Living Environments in London (ENABLE London) project is a natural experiment which aims to establish whether physical activity and other health behaviours show sustained changes among individuals and families relocating to East Village (formerly the London 2012 Olympics Athletes' Village), when compared with a control population living outside East Village throughout.

**Participants:**

Between January 2013 and December 2015, 1497 individuals from 1006 households were recruited and assessed (at baseline) (including 392 households seeking social housing, 421 seeking intermediate and 193 seeking market rent homes). The 2-year follow-up rate is 62% of households to date, of which 57% have moved to East Village.

**Findings to date:**

Assessments of physical activity (measured objectively using accelerometers) combined with Global Positioning System technology and Geographic Information System mapping of the local area are being used to characterise physical activity patterns and location among study participants and assess the attributes of the environments to which they are exposed. Assessments of body composition, based on weight, height and bioelectrical impedance, have been made and detailed participant questionnaires provide information on socioeconomic position, general health/health status, well-being, anxiety, depression, attitudes to leisure time activities and other personal, social and environmental influences on physical activity, including the use of recreational space and facilities in their residential neighbourhood.

**Future plans:**

The main analyses will examine the changes in physical activity, health and well-being observed in the East Village group compared with controls and the influence of specific elements of the built environment on observed changes. The ENABLE London project exploits a unique opportunity to evaluate a ‘natural experiment’, provided by the building and rapid occupation of East Village. Findings from the study will be generalisable to other urban residential housing developments, and will help inform future evidence-based urban planning.

**Strengths and limitations of this study**

- The ENABLE London project is a controlled cohort study, evaluating a natural experiment to examine the effect of moving into social, intermediate and market rent accommodation in East Village (formerly the London 2012 Athletes' Village), on physical activity, health and well-being indicators.
- In total, 1497 participants (1278 adults and 219 children) from 1006 households located in Newham and Greater London have been recruited.
- Two-year follow-up of those in social housing is largely complete with 62% participation and where 57% have moved to East Village*.* Follow-up of those seeking intermediate and market-rent accommodation will continue to December 2017.
- The data set includes demographic, lifestyle, health and well-being indicators, measures of anthropometry (including bioimpedance), objective measures of physical activity combined with individual Geographical Positioning System data, and Geographical Information System-determined environmental measures of the local area.
- East Village provides family-sized accommodation. While the study is well powered to detect change in physical activity associated with moving to East Village in adults, too few children moved in to establish change in younger participants.

## Introduction

Low physical activity is widespread and poses a serious public health challenge in the UK and worldwide.^1^ The need to increase population levels of physical activity is recognised in current health policy recommendations.^2^ ^3^ However, interventions to increase physical activity levels, particularly community-wide interventions, have shown limited effects, which are poorly maintained in the longer term.^4^ ^5^ There has been increasing interest in whether the built environment, especially in urban settings, might be a key constraint that limits opportunities for physical activity.^6^ However, there is very limited high-quality experimental evidence examining the influence of change in the built environment on physical activity.^6^ ^7^ The ENABLE London project has been established to address this issue, by providing evidence from the investigation of a natural experiment examining whether changing the built environment can increase physical activity levels, as well as indicators of physical and mental health, in the general population. This question has important public health relevance, as small shifts in population levels of physical activity, in addition to other markers of health and well-being, appear to have an appreciable impact on health-related outcomes.^8^

The ENABLE London study takes advantage of the natural experiment provided by the rapid change of brown-field land in the London Borough of Newham, to create a novel built environment for public use and occupancy (namely ‘East Village’ E20, formerly the London 2012 Olympic Games Athletes' Village). East Village is a planned mixed-use residential neighbourhood development, incorporating commercial, retail, educational and transportation resources, with 1439 housing units for market rent, 704 intermediate units and 675 households for social rent. Specific activity permissive features designed to encourage physical activity include improving access to open land and parkland, unrivalled transport links, and active travel options (including extensive walking and cycling paths), design features of the local environment (such as street furniture, provision and arrangement of pedestrianised space, public space aesthetics, secure bicycle parking) and the provision of new formal cycling and walking facilities in the Queen Elizabeth Olympic Park such as the VeloPark, and cycle paths which extend into the Lee Valley and connect to the London Cycle Network.^9^ ^10^ A local school, Chobham Academy, is within walking distance and provides schooling for all 3–19 years. Retail outlets were planned within easy walking distance for everyday use (creating plazas at ground level within dedicated areas of East Village).^9^ ^10^ Moreover, East Village is within the close proximity of Westfield Stratford City—Europe's largest urban shopping centre. Restriction of resident car parking (where less than a sixth of homes have a designated parking space) combined with improved public transport links is designed to encourage local residents to adopt active modes of transport.^9^ ^10^

ENABLE London participants moving to East Village will be directly exposed to the new social and built environment, and its active design features, in the follow-up phase of the study. Participants who were seeking to move to East Village but remain in their place of origin (largely in East London) or move elsewhere will act as controls. The inclusion of occupants of social, intermediate and market rent accommodation will allow the study to examine the effects of the East Village environment on individuals from widely differing social origins, and to establish whether the effects differ by socioeconomic group.

The study evaluates a natural experiment, based on the provision of high-quality homes located in a neighbourhood specifically designed to encourage healthy, active living for people in the social, intermediate and market rent sectors. ENABLE London is one of a handful of studies of its type,^11–13^ the findings from which could help to inform future urban residential housing developments. While the East Village development is unique in origin, scale and spread, its impact should be generalisable to other major inner city conurbations, given the replication of this type of high-density housing in other settings. This is important given global calls to create more compact higher density cities.^14^

## Cohort description

### Participants

The baseline population for this cohort were individuals and families who were seeking or applied for either social, intermediate or market rent accommodation in East Village. Most lived in East London, particularly the London Borough of Newham. Recruitment and baseline data collection were carried out between 1 January 2013 and 31 December 2015, before participants moved to East Village. In total, 1497 individuals (1278 adults, 219 children) were recruited from 1006 households. East Village did not attract as many families as anticipated, which explains the limited number of children recruited to participate in the study. Hence, only adults are considered further.

### Recruitment

**Figure 1:**
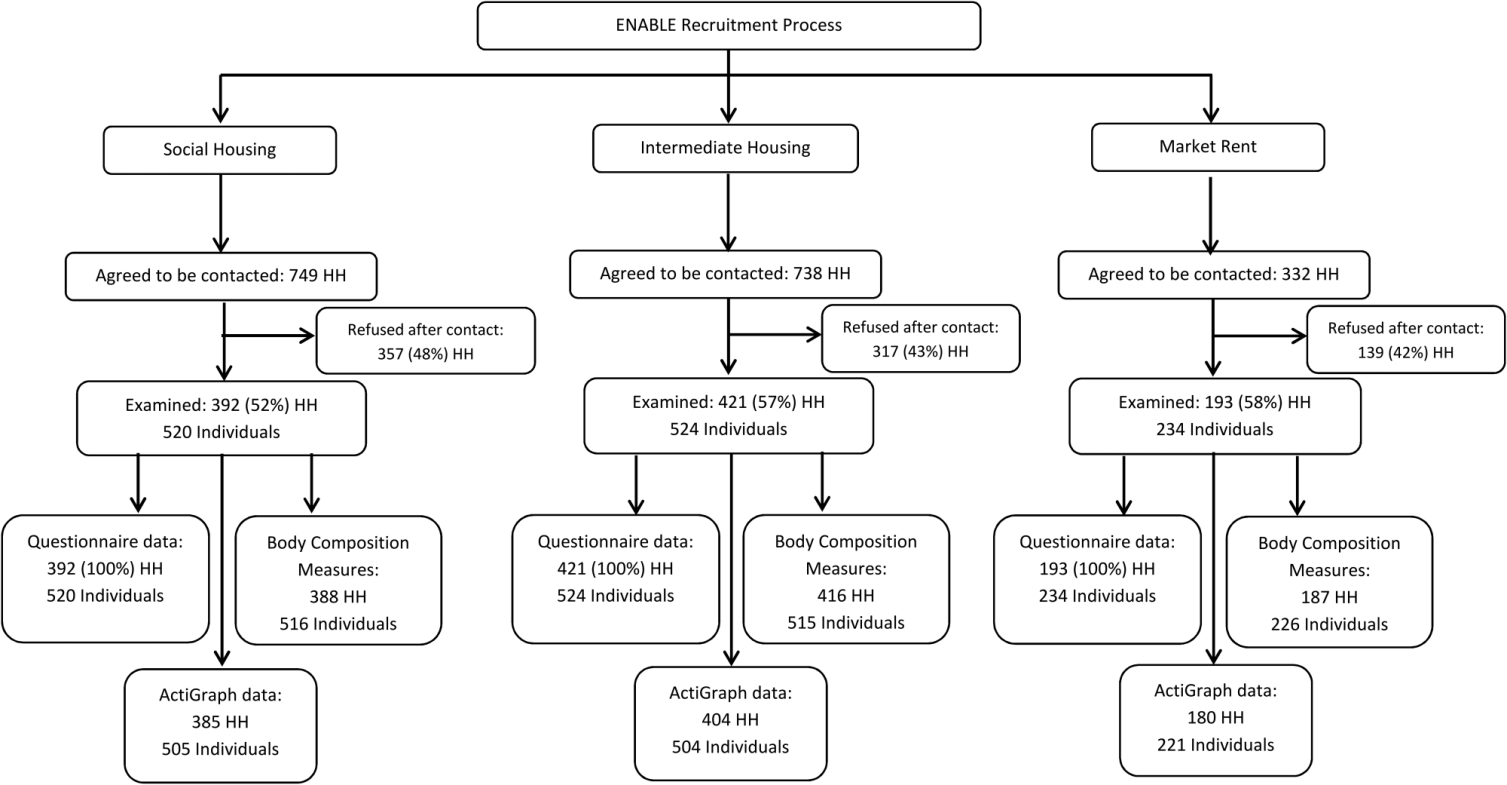
Flow diagram of adult participation by housing sector.

There were three distinct phases of recruitment for the different housing sectors: 392 households from the social sector were initially recruited between January 2013 and May 2014, 421 households seeking intermediate accommodation between July 2013 and November 2014 and 193 seeking market rent accommodation between September 2014 and December 2015; lower numbers recruited within the market rent sector reflected limitations on the extent and duration of access to applicants for accommodation. Recruitment processes for those in social housing were slightly different compared with other housing sectors. The East Thames Group was primarily responsible for recruiting participants in social housing, whereas the ENABLE London team (in association with Triathlon Homes and Get Living London) recruited participants from the other housing sectors. A flow diagram (figure 1) summarises recruitment and participation by housing sector. Of those who agreed to be contacted, participation rates were just over half in the social sector (52%), but higher in those seeking intermediate and market rent accommodation (57%, 58%, respectively).

### Data collection

Baseline and 2-year follow-up of study participants are being carried out at the participants' home (or at location convenient to the participant). Data items collected in the ENABLE London study at baseline and follow-up are listed in box 1 and summarised below.

Box 1 **Summary of data items collected at baseline and 2-year follow-up of the ENABLE London study**

Physical activity and location data

- ActiGraph GT3X+accelerometer worn for 1 week (ActiGraph LLC, Florida, USA)
- QStarz BT-100XT GPS travel recorder worn for 1 week (QStarz International Co, Taiwan)
- GIS Ordinance Survey mapping of place of residence at baseline and 2-year follow-up to provide measures of land-use mix, street connectivity, residential density, walkability and connectivity indices

Anthropometry

- Height measured to the last complete millimetre (Leicester Stadiometer, Seca, Birmingham, UK).
- Weight measured to the last complete 0.1 kg using an electronic digital scale, and fat mass (kg), fat free mass (kg), muscle mass (kg) measured by leg-to-leg bioimpedance (Tanita SC-240 Body Composition Analyzer, Tanita, Tokyo, Japan)
- BMI calculated as weight/height squared in kg/m^2^

Questionnaire data

- Demographics including date of birth, gender and ethnicity of participant
- Number of people living in the household, relationships, type of accommodation, household features (including lifts, stairs, garden), type of tenure, duration at current property, vehicles owned and dog ownership
- Qualifications, employment status and job title of adult participants (based on Census 2011 questions)
- Method of travel to work/place of study and daily commuting times
- Household income either as weekly or monthly amounts (based on National Evaluation of Sure Start income questions)
- Perception of general health, self-report of health problems (based on Census 2011 questions) and effects on mobility
- Health outcomes including assessments of mobility, self-care, usual activities, pain/discomfort, anxiety/depression and overall perception of health on a scale from 0 to 100 (using EuroQol EQ-5D questions)
- Satisfaction scores including perception of overall levels of satisfaction, feeling happy and anxious on a scale from 0 to 10 (based on questions used in the Integrated Household Survey 2011), and further assessment of anxiety and depression based on the Hospital Anxiety and Depression Scale
- Current and previous smoking status, and current alcohol consumption (using Health Survey for England questions)
- Perceptions of the local area/neighbourhood, including transport, leisure activities, vandalism, litter, traffic, attractiveness and safety, as well as assessment of social participation, support, cohesion and trust
- Type of activities carried out and frequency of carrying out vigorous, moderate, walking, sitting activities in the last 7 days (based on the IPAQ short questionnaire)
- Cost of activities including membership fees, vouchers received, equipment bought to do physical activity
- Attitudes to exercise
- TV and computers/screen time assessment
- Eating and sleeping

#### Physical activity level/pattern and location

Objectively measured physical activity was the primary outcome, and was assessed over 7 days using hip-mounted ActiGraph GT3X+accelerometers, combined with assessment of physical activity location using Geographical Positioning System (GPS) travel recorders (Qstarz BT-1000XT). Accelerometers provided daily measures of steps, light and moderate to vigorous physical activity (MVPA—both overall and in 10 min bouts, in accordance with UK physical activity recommendations).^3^ Simultaneous use of ActiGraph accelerometers and GPS Travel recorders allows walking components of physical activity, as well as indoor and outdoor activities, to be identified, using methods previously described by the investigators.^15^ ^16^ In addition, GPS data allow the geographical location at which different levels of physical activity occurs (from sedentary to vigorous, using established cut-offs in accelerometer data), at baseline and follow-up, to be identified. Together, these measures allow accelerometry data to be interpreted in depth, allowing the nature and location of recorded activities, particularly active forms of transport, such as walking and cycling, to be identified. Moreover, it allows the contribution of active transport local to place of residence to be quantified and compared between those living in East Village and control areas.

#### Environmental exposures

A Geographical Information System (GIS) was used to extract objective data on features of the local environment. In combination with ActiGraph and GPS data from study participants, this has allowed the location of where different levels of physical activity have been carried out (including high and low levels of activity), to be accurately identified. This method has been previously used by the investigators to establish the important contribution of walking to school and location (including land use type) to MVPA levels in children.^16^ ^17^ In the present study, a number of data sources are being used to identify environmental and activity permissive features within East Village and control areas, including Ordnance Survey (OS) MasterMap, Integrated Transport Network (ITN) and Transport for London (TfL) sources, Olympic Delivery Authority and Local Authority data as well as other printed an online resources. In particular, OS data are being used to derive indices, such as land-use mix, street connectivity, residential density, walkability and connectivity indices, including walking distance to particular features of the built environment, including green space.^18^

#### Anthropometric measurements

Height was measured to the last complete millimetre with a portable stadiometer at baseline and follow-up (Leicester Stadiometer, Seca, Birmingham, UK). Weight and leg-to-leg bioimpedance were assessed using an electronic Tanita SC-240 body composition analyser (Tanita, Tokyo, Japan) to provide measures of fat mass (kg) and fat free mass (kg); body mass index (BMI) was calculated as weight/height^2^ (kg/m^2^). In total, eight Leicester stadiometers and Tanita SC-240 body composition analysers were used to measure participants. The Tanita devices were operated using factory default settings and were regularly checked in accordance with recommended review procedures.

#### Questionnaire data

Questionnaires were converted into electronic format using SNAP Surveys software (V.11, SNAP Surveys, London, UK), and completed by study participants using dedicated laptops. Questionnaires used established validated methodologies to collect detailed information on patterns and types of activity local to place of residence. In particular, the ‘Neighbourhood Physical Activity Questionnaire’ provides data to examine walking within the neighbourhood,^19^ and the ‘Neighbourhood Environment Walking Scale’ (NEWS) perceptions of the neighbourhood environment.^20^ ^21^ Information on self-defined ethnic origin (based on the Census, 2011) and a range of social markers were recorded (including employment status, income, duration and location of work), together with home address and postcode of residence, allowing GIS-determined distance to local amenities to be measured. Questions about general health/health status,^22^ well-being, anxiety and depression, including clinical and subclinical forms of assessment suitable for use in community settings, have also been used.^23–26^ Physical activity was assessed using an adaptation of the short-form, self-reported International Physical Activity Questionnaire (IPAQ),^27^ to provide perceived levels of physical activity in addition to objective measures. Adults are asked about attitudes to physical activities (including sedentary, such as screen-time, and physically active forms) and factors which influence their physical activity behaviour. Participants are asked about perceived personal, social and environmental influences on physical activity, their use of recreational space (particularly walkways and cycle paths) and facilities in their residential neighbourhood (including costs incurred). Participants are also asked about the availability, accessibility (method of travel and journey times) and usage of local amenities (walkways, cycle paths, parks, swimming pools, etc); their perceptions of the safety of these amenities and the degree to which they permit their child independent or supervised use. The questionnaire also includes sections to ascertain levels of social participation, support, cohesion and trust.^28^ These items are particularly relevant to gauge how use and perceptions of the local area by others impact on individual use and how this might differ from objectively measured features of their neighbourhood.

#### Qualitative data

In addition to the rich quantitative data, focus groups among study participants who have and have not moved to East Village have been carried out to identify issues of importance, particularly about perceptions and use of their local environment. GIS and GPS data are also being combined with qualitative spatial narratives among study participants. These narratives use individual participant maps of travel patterns to provide context of use, that is, reasons and purpose of travel and to tailor interviews to investigate how the built environment has influenced individual patterns of behaviour.

*Ethical approval:* The study was approved by the City Road and Hampstead Ethical Review Board (REC reference number 12LO1031); all participants gave written, informed consent.

### Characteristics of study participants

Table 1 summarises baseline characteristics of adult participants by housing sector; the small number of children were recruited from the social sector and have not been included further. Participants from social housing were older, had a higher number of participants per household, with greater representation of females (73%) and those of Black and Asian ethnic origin. Participants seeking intermediate and market-rent housing were younger, more equally gender balanced (48%, 44% female, respectively), and had higher representation of whites compared with other ethnic groups. The proportion of those reporting poorer general health was higher among those in social housing, compared with other housing sectors (table 1). Moreover, the percentage reporting medium to high levels of satisfaction with life was higher among those seeking market rent (81%) and intermediate (78%) accommodation, compared with those in the social sector (68%—table 1). Two-year follow-up of those in the social sector began in January 2015 and is now largely complete; follow-up of those seeking intermediate or market rent accommodation will continue to December 2017.

**Table 1:** Baseline demographic, self-reported health status and local environment perceptions of ENABLE London adult participants, by housing sector

	Social housing	Seeking intermediate housing	Seeking market rent housing	All participants
Number of adults	520	524	234	1278
Number of adults/household	1.3	1.2	1.2	1.3
Median age (IQR)	36.6 (27.3, 44.2)	29.8 (26.0, 34.8)	27.7 (24.4, 33.1)	31.1 (25.7, 39.5)
Female (%)	379 (72.9)	249 47.5	103 44.0	731 57.2
Ethnicity (%)				
White	96 (18.5)	358 (68.3)	163 (69.7)	617 (48.3)
Black	251 (48.3)	55 (10.5)	17 (7.3)	323 (25.3)
Asian	108 (20.8)	77 (14.7)	29 (12.4)	214 (16.7)
Other	65 (12.5)	34 (6.5)	25 (10.7)	124 (9.7)
Employment status (%)*				
Employed	252 (48.8)	492 (93.9)	204 (87.2)	948 (74.4)
Economically inactive	264 (51.2)	32 (6.1)	30 (12.8)	326 (25.6)
NS-SEC (Employed only) (%)				
Higher managerial, administrative and professional	61 (24.2)	375 (76.2)	155 (76.0)	591 (62.3)
Intermediate occupations	62 (24.6)	79 (16.1)	38 (18.6)	179 (49.0)
Routine and manual occupations	125 (49.6)	34 (6.9)	11 (5.4)	170 (24.2)
Unclassified	4 (1.6)	4 (0.8)	0 (0.0)	8 (4.5)
General health status (Census) (%)				
Very good	140 (26.9)	153 (29.2)	72 (30.8)	365 (28.6)
Good	253 (48.7)	310 (59.2)	140 (59.8)	703 (55.0)
Fair	103 (19.8)	58 (11.1)	18 (7.7)	179 (14.0)
Bad	19 (3.7)	2 (0.4)	4 (1.7)	25 (2.0)
Very bad	5 (1.0)	1 (0.2)	0 (0.0)	6 (0.5)
HADS-anxiety (%)†				
Normal	332 (65.2)	369 (71.0)	148 (64.1)	849 (67.4)
Borderline	97 (19.1)	94 (18.1)	60 (26.0)	251 (19.9)
Abnormal	80 (15.7)	57 (11.0)	23 (10.0)	160 (12.7)
HADS-depression (%)‡				
Normal	316 (65.3)	413 (81.0)	194 (85.5)	923 (75.6)
Borderline	110 (22.7)	76 (14.9)	27 (11.9)	213 (17.4)
Abnormal	58 (12.0)	21 (4.1)	6 (2.6)	85 (7.0)
Satisfaction with life (%)§				
Very low	51 (10)	22 (4)	10 (4)	83 (7)
Low	118 (23)	95 (18)	34 (15)	247 (19)
Medium	185 (36)	308 (59)	156 (67)	649 (51)
High	164 (32)	98 (19)	33 (14)	295 (23)
Local perceptions—enjoy living in the local area (%)				
Strongly agree	83 (16.0)	149 (28.4)	57 (24.4)	289 (22.6)
Agree	192 (36.9)	212 (40.5)	110 (47.0)	514 (40.2)
Neither	111 (21.3)	89 (17.0)	45 (19.2)	245 (19.2)
Disagree	78 (15.0)	62 (11.8)	16 (6.8)	156 (12.2)
Strongly disagree	56 (10.8)	12 (2.3)	6 (2.6)	74 (5.8)

*Four missing responses.

†Eighteen missing responses.

‡Fifty-seven missing responses.

§Four missing responses.

HADS, Hospital Anxiety and Depression Scale; NS-SEC, National Statistics Socioeconomic Classification.

## Findings to date

The ENABLE London study has recruited participants from different housing sectors (table 1). Baseline data have previously shown that those in social housing were less likely to report enjoying living and walking in their local neighbourhood, that their local area is attractive to look at and that they have good local transport and leisure services.^29^ They were also more likely to report problems with vandalism and litter in their local area, as well as having greater concerns over crime and safety, compared with the other housing types.^29^ Too much traffic was reported as a problem across all housing sectors.^29^ Two focus groups among those in the social housing sector have been completed to date; one in a group who have moved to East Village and another in those who have not moved to East Village (7–9 participants in each). Among those who had moved, East Village was recognised as a safe, clean, spacious environment, with good local facilities, including public transport, which encouraged walking activities. However, the cost of living was high, with few shops, particularly super markets, serving their income range, making it more difficult to save. The cost of living was also reported as a problem among the non-movers, which limited opportunities for physical activity in the local area. These themes will be explored in further focus group among study participants in other housing sectors.

Compliance with wearing the ActiGraph physical activity monitor, defined as 9 hours wear for at least 4 days, was good with nearly two-thirds recording adequate wear (66%) in social households, 84% and 89% among those seeking intermediate and market rent housing, respectively. Objective measures of physical activity showed lower levels of activity among those in social housing, with fewer daily steps, and less time spent in higher levels of activity (table 2). Time spent in 10 min bouts of MVPA (equivalent to just over 100 min/week) were well below current recommendations of 150 min/week in all sectors, and markedly lower among those in social housing.^3^ How these objective measures of physical activity relate to GIS-derived measures of walkability will be an early focus of our work,^30^ allowing validation of a walkability index developed in an American setting, to be objectively validated within a European context, by combining GIS, GPS and ActiGraph data recorded at an individual level.^18^ The need to further understand the relationship between the physical environment and activity within European settings has recently been highlighted.^31^ In addition, measures of anthropometry suggest higher levels of adiposity, including measures of BMI, obesity (defined as ≥30 kg/m^2^), fat mass and fat mass-derived levels of obesity (defined as ≥30% body fat in women, and ≥25% body fat in men),^32^ among adults in the social sector compared with other housing sectors, with similar levels among those seeking intermediate and market rent accommodation (table 2). However, the influence of age, gender and ethnicity on these differences is yet to be determined.

**Table 2:** Baseline objective measures of physical activity and anthropometry of ENABLE London adult participants, by housing sector

	Social housing	Seeking intermediate housing	Seeking market rent housing	All participants
Daily physical activity	505	504	221	1230
Compliance*	66%	84%	89%	78%
Number with compliant PA data	336	421	197	954
Steps/day	7803 (3303)	9684 (2924)	9337 (2990)	8950 (3190)
Time in light activity (min/day)†	175 (140, 212)	128 (101, 157)	117 (90, 156)	139 (106, 180)
Time in MVPA (min/day)	50 (26)	65 (23)	65 (25)	60 (26)
Time in 10 min bouts of MVPA (min/day)†	7 (1, 15)	21 (10, 34)	21 (12, 36)	15 (6, 30)
Registered time (min/day)	775 (82)	802 (72)	808 (69)	794 (77)
Anthropometry	516	515	226	1257
Height (m)	1.65 (0.09)	1.71 (0.10)	1.72 (0.10)	1.69 (0.10)
Weight (kg)†	70.9 (62.7, 84.1)	70.6 (61.8, 80.8)	72.8 (61.0, 80.3)	71.1 (61.9, 81.7)
BMI (kg/m^2^)†	26.3 (23.4, 30.5)	23.9 (21.9, 26.7)	23.8 (21.5, 25.8)	24.7 (22.2, 27.8)
Number obese based on BMI (%)‡	138 (26.7)	50 (9.7)	13 (5.8)	201 (16.0)
Fat mass (kg)†¶	22.8 (15.6, 31.2)	15.4 (11.1, 21.4)	14.8 (10.8, 19.9)	17.7 (12.6, 25.5)
Number obese based on fat mass (%)§¶	315 (61.4)	145 (28.8)	52 (23.1)	512 (41.3)

Mean and SD presented for normally distributed variables.

*Compliance defined as 9 hours/day for at least 4days.

†Non-normally distributed variables presented as median and IQR (lower quartile to upper quartile).

‡Obesity defined as BMI≥30 kg/m^2^.

§Obesity defined as ≥30% body fat in females, and ≥25% body fat in males.

¶Sixteen missing responses.

**Figure 2:**
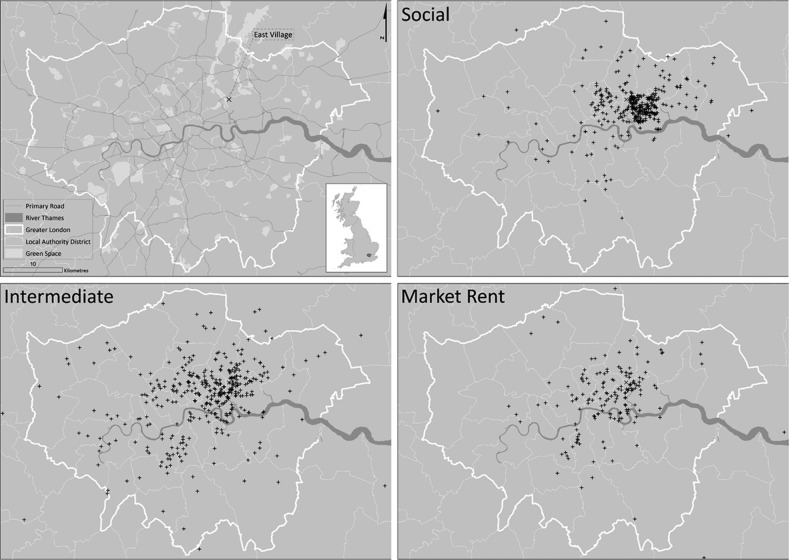
Baseline locations of social, intermediate and market rent households participating in the ENABLE London study.

Two-year follow-up of the cohort will provide the opportunity to examine whether indicators of health and well-being, perceptions of the local living environment and objective measures of physical activity and adiposity change on moving to East Village, compared with change observed among those who do not move to East Village. All analyses will allow for the hierarchical nature of the data, using multilevel models to take appropriate account of factors operating within subject, as well as at individual and household level in East Village and control areas. Models will be extended to examine whether any differences between the intervention and control areas are modified by age group, gender, ethnic group, social class, housing sector, proximity and accessibility to certain facilities. The extent to which changes in physical activity in those living in the Village can be directly attributed to the use of local facilities, and which facilities in particular, will be examined using data from questionnaire, GPS and GIS measures. Subsidiary objectives (such as change in weight and body fat) will be addressed using a similar analytical approach, but without the need to allow for replicates within participant; multilevel models will be extended to include binary as well as continuous outcomes. Time-dependent covariates that might affect absolute levels of physical activity such as weather should, by design, be balanced between the intervention and control area by examining study participants at similar times of year, but we will also explore linking Met Office weather data to directly control for weather. Further details of the analysis plan have been published.^30^ Follow-up of those in the social sector is largely complete, with 62% of the entire baseline cohort being seen to date; 57% have moved to East Village and 43% have not. Figure 2 shows the geographic home locations of study participants at baseline, which highlights the Newham focus among those in social housing, and greater London geographic diversity of participants seeking intermediate and market rent accommodation. Follow-up of the remaining cohort is likely to show a greater skew towards those who have moved to East Village, due to more focused marketing of intermediate and market rent accommodation. However, the study design is robust to some imbalance between the number of movers and non-movers.

Early priorities for the study will be to identify changes in physical activity and other health behaviours, well-being and perceptions of the environment between those that move and do not move to East Village, to understand their potential sociodemographic determinants and whether these differ across housing sectors. If change is observed, we will examine whether this can be attributed to specific features of the East Village built environment, identified objectively using GIS mapping and self-report measures of the area.

## Strengths and limitations

The building of East Village provided an important opportunity to evaluate a ‘natural experiment’ based on the major and focused change of an inner city urban built environment that has been specifically designed to encourage walking, cycling and healthy living. It is also unique because it involves residents from widely differing socioeconomic backgrounds. While many developments of this scale are underway or are being planned in cities globally, few have been (or are being) evaluated and most are less easily evaluated, given that the timescale of their development is much slower than in the case presented by East Village. The rapid occupancy of this development is a major strength, providing the opportunity for preassessment and postassessment, and to compare 2-year change in health outcomes among those who do and do not move to East Village. The different housing tenures within East Village also allow for the evaluation of socioeconomic position as an effect modifier, as the impact of the built environment may vary by socioeconomic position. The focus on increasing levels of accessible and low-cost forms of physical activity will be particularly relevant to individuals and households of lower socioeconomic status and has the potential to inform efforts to reduce health inequalities.^33^ This will allow us to investigate whether the built environment can favourably influence higher levels of physical activity (particularly walking and cycling), as well as reducing time in sedentary activities, particularly among low-income groups with fewer opportunities for recreational activities.^33^ Moreover, the colocation of this diverse population of differing housing tenures to one community also constitutes a social experiment, providing opportunities for residents to observe and learn from the behaviour of others.^34^

The ENABLE London cohort was predicated on recruiting 1200 adults from 1200 households, and has succeeded in recruiting 1278 adults from 1006 homes. Given the modest imbalance between movers and non-movers, the compliance and follow-up rate observed to date, the study is powered to detect a 750 step change (0.3 SD) at 90% power and with a probability of 0.01 among those who move to East Village. The initial aim was to recruit similar numbers of adults and children (aged 8 or more years), especially as most of the accommodation in East Village is family-sized (ie, 2 bedrooms or more). Although this was partially achieved in the social sector (with 209 children, largely due to the allocation to families in need of rehousing), the baseline demographic of those seeking intermediate and market rent accommodation had an adult focus. This reflects the demography of those who have chosen to move to East Village, which is heavily skewed towards young professionals. This demographic profile was an unexpected outcome of the development, which was purposely designed for family occupancy. The study remains well powered to detect any potential change in adult physical activity.

In terms of the representativeness of the ENABLE London cohort, we have compared our physical activity data to a nationally representative study, Health Survey for England 2008,^35^ which used a similar methodology, that is, the same waist-worn accelerometer (ActiGraph), worn for an equivalent wear time (1 week). Adults aged 16–34 years from this study recorded 40 min/day in MVPA, of which 15 min was in 10 min bouts. Our baseline data suggest comparable levels of activity among those of a similar age in the social sector, with 47 min of daily MVPA, 7 min in bouts (with an IQR between 1 and 15 min), but higher levels among those in the intermediate and market rent sectors with 65 min of MVPA and >20 min recorded in bouts. While this suggests differences in baseline physical activity levels across the housing sectors in the ENABLE London cohort, there was no evidence of a trend across other social markers (ie, income groups) in the Health Survey for England study. In terms of geographic patterns in physical activity, reanalysis of Health Survey for England (2012) data did not suggest that self-reported higher levels of physical activity in London were unduly higher or lower compared with other Government Office Regions.^36^

Specific features of the East Village development that could influence health and well-being (such as use of pathways, cycle paths, links to public transport, open spaces and leisure facilities) are (or could be) features of many built environment developments. The GIS measures will identify the availability of facilities, while the GPS will specifically allow the use of such facilities to be identified (including frequency of use, and time of day) and related to type of use (for leisure, work, etc) and objectively measured duration and intensity of physical activity associated with their use. Furthermore, questionnaire data combined with qualitative focus groups and spatial narratives will allow us to investigate how perception of the built environment influence travel patterns. Hence, findings from this study will have substantial potential for wider generalisability to other inner city major conurbations. An ultimate goal of the project is to identify evidence-based design features of the built environment that encourage physical activity and improve health behaviours. It is hoped that the identification of these environmental features will provide architects, urban designers and planners with evidence-based urban design elements, which are required for future developments.
